# A Systematic Review and Meta-Analysis of 19 Randomized Controlled Trials of Iguratimod Combined With Other Therapies for Sjogren’s Syndrome

**DOI:** 10.3389/fimmu.2022.924730

**Published:** 2022-07-28

**Authors:** Liuting Zeng, Qi He, Kailin Yang, Wensa Hao, Ganpeng Yu, Hua Chen

**Affiliations:** ^1^Department of Rheumatology and Clinical Immunology, Peking Union Medical College Hospital, Chinese Academy of Medical Sciences & Peking Union Medical College, National Clinical Research Center for Dermatologic and Immunologic Diseases (NCRC-DID), Key Laboratory of Rheumatology and Clinical Immunology, Ministry of Education, Beijing, China; ^2^People's Hospital of Ningxiang City, Ningxiang City, China; ^3^Key Laboratory of Hunan Province for Integrated Traditional Chinese and Western Medicine on Prevention and Treatment of Cardio-Cerebral Diseases, Hunan University of Chinese Medicine, Changsha City, China

**Keywords:** iguratimod, Primary Sjogren’s syndrome, systematic review, meta-analysis, randomized controlled trials

## Abstract

**Objective:**

To explore the efficacy and safety of Iguratimod intervention in Primary Sjogren’s syndrome (pSS).

**Methods:**

Many databases were searched to collect the RCTs. Three independent reviewers extracted data and assessed the quality of the studies based on the Cochrane Handbook. The statistical analysis was done by RevMan 5.3 and STATA. The quality of evidence was evaluated by GRADE tool.

**Results:**

Twenty-nine RCTs with 2258 participants were included in this review. The meta-analysis shows that: iguratimod experiment group can reduce the ESSPRI score (WMD -1.93 [-2.33, -1.52], P<0.00001), ESSDAI score (WMD -1.39 [-1.81, -0.98], P<0.00001), Schirmer’s test (WMD 1.77 [0.85, 2.70], P=0.0002), RF (WMD -5.78 [-7.59, -3.97], P<0.00001), and decrease the ESR level (WMD -7.05 [-9.84, -4.26], P<0.00001). Meanwhile, the summary result showed the addiction of Iguratimod may not increase the adverse events. The adverse events were mainly gastrointestinal discomfort, abnormal liver function, and rash and itching. The quality of evidence of adverse events was moderate. Referring to minimal clinically important difference (MCID), the improvement of ESSPRI is clinically significant, and the improvement of ESSDAI for patients older than 60 years old may be clinically significant.

**Conclusion:**

Based on current evidence, iguratimod can effectively reduce ESSPRI score, ESSDAI score, Schirmer’s test score and decrease systemic inflammatory response (such as ESR level and RF level) without increasing the probability of adverse events. The recommended course of treatment is at least 12 weeks.

**Systematic Review Registration:**

https://www.crd.york.ac.uk/prospero/display_record.php?, identifier CRD42020220770.

## 1 Introduction

Primary Sjogren’s syndrome (pSS) is a chronic inflammatory autoimmune disease that mainly involves exocrine glands (especially salivary glands and lacrimal glands), and is accompanied by chronic autoimmune diseases with visceral involvement [(1); Thorne and Sutcliffe 2005 (2)]. Its clinical manifestations are mainly dry eyes (keratoconjunctivitis sicca) and dry mouth (xerostomia) (2). The pathogenesis of Sjogren’s syndrome is still unclear. Pathology and serology, as well as the (albeit weak) association with HLA alleles, have shown that viral infection of the salivary glands leads to local cell death and the release of tissue autoantigens leading to the activation of autoreactive T cells and B cells. This ultimately leads to lymphocytic infiltration of the glands (lacrimal and salivary glands) (3–5). According to the patients with different levels of symptoms, the current treatment and management of primary Sjogren’s syndrome mainly include non-steroidal anti-inflammatory drugs, glucocorticoids, immunosuppressants, and biological inhibitors. When multiple organs are involved, a combination of the above drugs is often required (6–8), but the current treatment effect is still not good. Although the current comprehensive management strategies for pSS are entering the stage of biological targeted therapy, their efficacy in pSS is still imprecise. To date, two large randomized controlled trials (RCTs) have been conducted to evaluate the efficacy of rituximab in pSS (9, 10). The Devauchelle-Pensec et al. study included 120 patients to evaluate the efficacy of a single course of rituximab. However, the study did not meet the primary endpoint: at 24 weeks, the visual analogue scale (VAS, 0-100mm) did not decrease by 30mm in any two of the four measures (dryness, pain, fatigue and global assessment of disease activity). In addition, open-label studies of abatacept in pSS did not meet the primary efficacy outcome of improvement in ESSDAI score from baseline to week 24 (11, 12). At present, there is still no definite therapeutic drug for the clinical treatment of PSS. Iguratimod has shown an effect in the treatment of pSS, but different results have been reported in RCTs. Iguratimod, as a new anti-rheumatic drug (DMARDs) to improve the condition, is mainly used in rheumatoid arthritis in China and Japan (13–15). Iguratimod has a comprehensive immunomodulatory effect whether in cellular immunity (T cell) (16) or humoral immunity (B cell) (17). In addition, Iguratimod also has a powerful anti-inflammatory effect, such as TNF-α and interleukin factors (IL-6, IL-8, IL-17) and other inflammatory factors (18, 19).

Given that T cells and B cells play an important role in the pathogenesis of pSS (8), Iguratimod is a promising treatment for pSS. The current study also shows that Iguratimod can improve the EULAR Sjogren’s Syndrome Activity Index (ESSDAI) score and EULAR Sjogren’s Syndrome Reporting Patient Index (ESSPRI) score, and reduce the patient’s immune globulin, ESR, rheumatoid factor (RF) and B cell percentage (20). At present, some RCTs of Iguratimod in the treatment of pSS have been carried out clinically. A previous systematic review and meta-analysis evaluated the efficacy and safety of Iguratimod in the treatment of PSS, but the included RCTs were not enough and the quality was not high, and they did not include RCTs related to complications of PSS. Therefore, the conclusion may have limitations (21). Since Iguratimod was approved as a clinical recommended drug in China, a large number of RCTs have emerged, which may correct the results. Therefore, this study would conduct a systematic review and meta-analysis of Iguratimod combined with other therapies in the treatment of pSS to assess the efficacy and safety of Iguratimod in patients with pSS compared with other DMARDs.

## 2 Materials and Methods

### 2.1 Protocol

This systematic review and meta-analysis were conducted strictly in accordance with the protocol registered in PROSPERO (CRD42020220770) and PRISMA-guidelines (see **Supplementary Materials**).

### 2.2 Literature Search Strategy

The Chinese Science and Technology Periodical Database (VIP), Web of Science, EMBASE, Medline Complete, Pubmed, ClinicalTrials, Chinese Biomedical Database (CBM), Wan Fang Database, the China National Knowledge Infrastructure Databases (CNKI), Cochrane Library were searched to collect clinical studies on the treatment of Sjogren’s syndrome by Iguratimod. The retrieval time is up to 1st April 2022. The search formula of Pubmed and EMBASE is shown in **Table S1** as an example. When searching the database, the constructed search formula is input to obtain search results.

### 2.3 Selection Criteria

#### 2.3.1 Participants

Patients who have been clinically diagnosed as primary Sjogren’s syndrome, without limitation to gender, age, course of disease, etc.

#### 2.3.2 Intervention and Control

The trial group received the treatment of iguratimod or combined with iguratimod on the basis of the control group. The control group received placebo treatment or conventional therapy.

#### 2.3.3 Outcomes

(1) Primary outcome is Disease activity score (ESSPRI and ESSDAI). (2) Secondary outcomes are Schirmer’s test and Inflammation related indicators [ESR, CRP, rheumatoid factor (RF)]. (3) Adverse events. The minimal clinically important difference (MCID) of ESSDAI is decrease≥3 points, the MCID of ESSPRI is decrease≥1 point, and the MCID of Schirmer’s test is increase≥ 5mm (22).

#### 2.3.4 Study Design

Randomized controlled trial, with no limitations to publication time, language, quality and publication status.

#### 2.3.5 Exclusion Criteria

(1) Adolescents (under 18 years of age); (2) the participant is not human; (3) Non-original research literature. (4) Studies with only one author will be excluded, as RCTs often require multi-investigator collaboration.

### 2.4 Literature Screening, Data Extraction and Quality Assessment

Two researchers independently screened the literature, extracted data and cross-checked. If there is a disagreement, the decision will be made through discussion and negotiation by all three reviewers. When selecting documents, the title and abstract are read first. After excluding irrelevant documents, the remaining documents will be further read through the full text to determine whether to include them. The data extraction content mainly includes: (1) Basic information of the included research: research title, first author, year of publication, etc.; (2) Baseline characteristics of the research object; (3) Important information such as outcome indicators. Two researchers independently used the risk of bias assessment tool based on the Cochrane Handbook to assess the methodological quality of the study (23), and when they disagree, the results were determined through discussion and negotiation by all three reviewers. The risk of bias assessment tool includes: Selection bias (random sequence generation and allocation concealment), Performance bias, detection bias, reporting bias, other bias.

### 2.5 Statistical Analysis

Review Manager 5.3 software was used for statistical analysis. If the statistical variable is a continuous variable, mean difference (MD) and 95% confidence interval (CI) are used as effect indicators. If it is a dichotomous variable, relative risk (RR) and 95% CI are used as effect indicators. Heterogeneity among studies was assessed using Cochrane’s Q and I^2^ statistic (24). The fixed effect model would be used when P>0.1, *I*2<50%. We would explore the reasons for heterogeneity, perform the subgroup analysis or use the random effect model when P<0.1, I2>50%. The publication bias was detected by STATA 15 with Egger method (continuous variable) and Harbord methods (dichotomous variable) for primary outcomes. P>0.1 is considered to have no publication bias.

### 2.6 Sensitivity Analysis

Sensitivity analyses were conducted by STATA 15 for all outcomes if they meet the following criteria:

1. the random effects model was utilized;
2. the results of the fixed effect model are inconsistent with that of the random effect model.

## 3 Results

### 3.1 Results of the Search and Description of Included Trials

**Figure 1:**
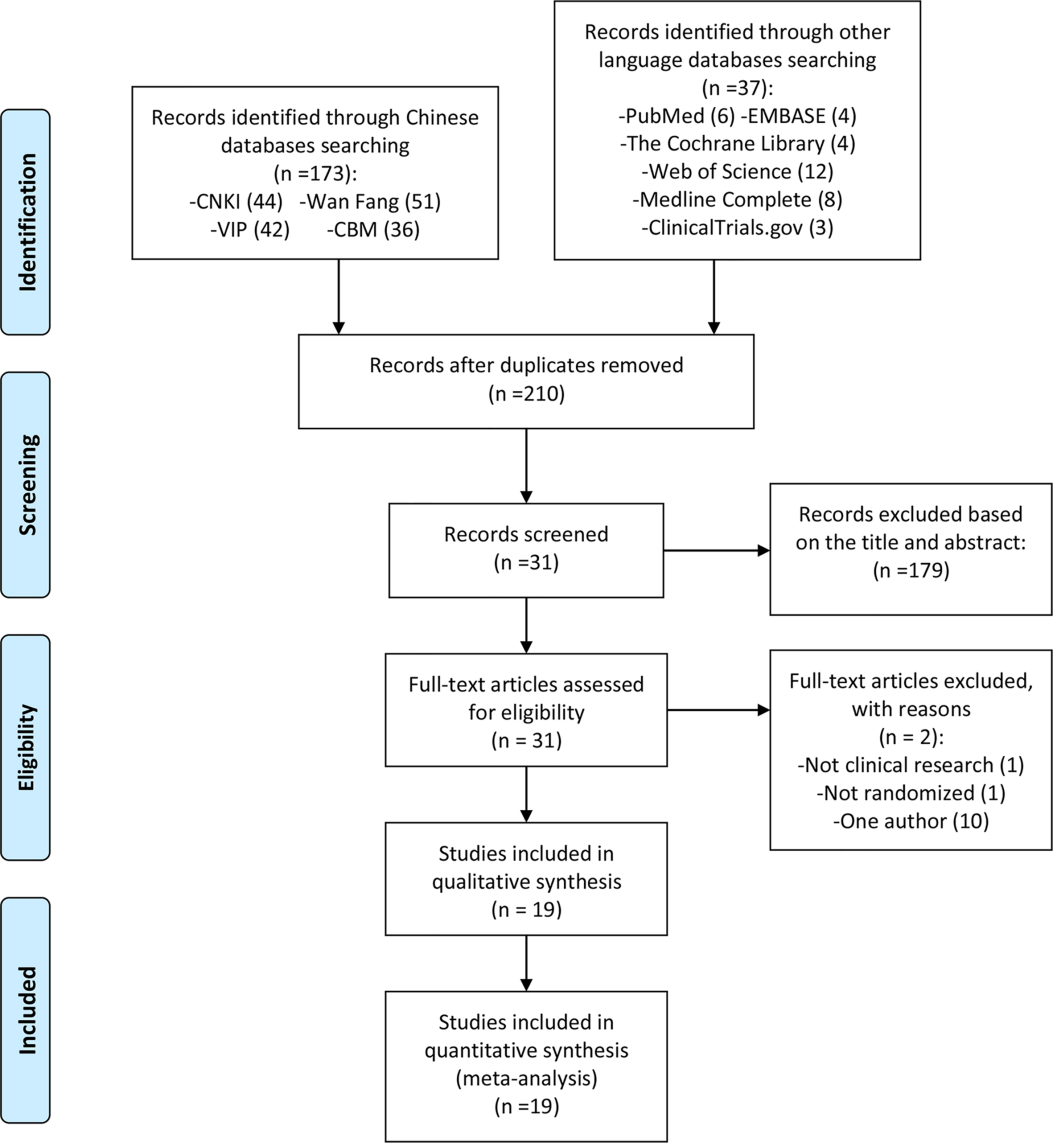
Flow diagram.

The total records identified through database searching and other sources were 210; 44 in CNKI, 51 in Wanfang, 42 in VIP, 36 in CBM, 6 in Pubmed, 4 in the Cochrane library, 3 in ClinicalTrials.gov, 4 in Embase, 8 in Medline, 12 in web of science. One hundred and seventy-nine (179) were excluded based on the title and abstract and 31 for more detailed evaluation. Two (2) of 31 records were excluded because they were not RCTs (20, 25), and 10 records were excluded because they only include one author (26–35) (**Figure 1**).

All RCTs comes from China. The scale of RCT is mostly between 40-80 participants. Only the control group of Shao et al. 2020 (36) used Placebo, Wang et al., 2019 (37), Xie et al. 2020 (38), Chen et al. 2022 (39) used Total Glucosides of Paeony + HCQ therapy, and the control group of the remaining studies used Conventional treatment. The ages involved in all RCTs are mostly between 25-70 years old, and the course of the disease is mostly within 30 years. The details of study characteristics are presented in **Table 1**.

**Table 1:** The characteristics of the included studies.

Study	Country	Sample size		Intervention		Relevant outcomes	Mean age (years)		Course of disease (years)		ESSPRI		Duration
		Trial group	Control group	Trial group	Control group		Trial group	Control group	Trial group	Control group	Trial group	Control group	
Jiang et al. 2020 (40)	China	25	25	Conventional treatment + Iguratimod 50 mg Q.d.	Conventional treatment [10 mg prednisone, 400 mg hydroxychloroquine (HCQ), new hydrochloride bromine ethyl Q.d.]	EULAR Sjögren’s syndrome patient-reported index (ESSPRI), ESSDAI, Schirmer’s test, Adverse events	29.3 ± 9.7	32.5 ± 11.5	7.5 ± 4.8	7.1 ± 6.6	7.36 ± 0.16	7.08 ± 0.15	12 weeks
Shao et al. 2020 (36)	China	44	22	Iguratimod 25mg B.i.d	Placebo	ESSPRI, ESR, ESSDAI, Adverse events	49.5 ± 12.3	48.2 ± 11.5	12.8 ± 14.1	10.3 ± 15.2	4.5 ± 2.7	4.2 ± 2.4	24 weeks
Jiang et al. 2016 (41)	China	30	30	Conventional treatment + Iguratimod 50 mg Q.d.	Conventional treatment [8 mg prednisone Q.d. +200 mg HCQ B.i.d]	Rheumatoid factor (RF), ESR, Adverse events	45.13 ± 12.11	46.33 ± 13.74	6.1 ± 2.3	4.9 ± 2.7	–	–	12 weeks
Wang et al. 2019 (37)	China	32	32	Iguratimod 25 mg B.i.d + Total Glucosides of Paeony 0.6g B.i.d. + HCQ 0.1g B.i.d	Total Glucosides of Paeony 0.6g B.i.d. + HCQ 0.1g B.i.d	ESSPRI, ESSDAI, Schirmer’s test, ESR, RF, Adverse events	66.8 ± 7.7	65.3 ± 8.2	0.5-11	0.7-10	6.6 ± 1.5	7.2 ± 1.4	12 weeks
Jiang et al. 2014 (42)	China	25	25	Conventional treatment + Iguratimod 25 mg B.i.d.	Conventional treatment [5-10 mg prednisone Q.d. +200 mg HCQ B.i.d+Bromoethylsine 16mg T.i.d]	ESSPRI, ESSDAI, Schirmer’s test, Adverse events	29.3 ± 9.7	32.5 ± 11.5	8-32	10-36	6.3 ± 1.5	7.1 ± 1.5	12 weeks
Bai et al. 2019(43)	China	30	30	Conventional treatment + Iguratimod 25 mg B.i.d.	Conventional treatment [8 mg methylprednisolone Q.d. +200 mg HCQ B.i.d]+ Leflunomide 50mg Q.d.	ESSPRI, ESSDAI, RF, ESR, Adverse events	43 ± 21	43 ± 10	6.1 ± 2.3	5.75 ± 2.92	6.4 ± 1.4	7.2 ± 1.4	12 weeks
Li et al. 2018(44)	China	34	34	Conventional treatment + Iguratimod 25 mg B.i.d.	Conventional treatment [8 mg prednisone Q.d. +200 mg HCQ B.i.d]	ESSPRI, RF, ESR, Adverse events	40.05 ± 3.16	40.02 ± 3.15	3.42 ± 0.26	3.15 ± 0.26	7.33 ± 1.01	7.24 ± 1.02	12 weeks
Li et al. 2020(45)	China	23	23	Conventional treatment + Iguratimod 25 mg B.i.d.	Conventional treatment [8 mg prednisone Q.d. +200 mg HCQ B.i.d]	ESSPRI, ESR, Adverse events	46.29 ± 1.24	46.38 ± 1.37	–	–	7.23 ± 1.01	7.34 ± 1.02	12 weeks
Xie et al. 2020(38)	China	38	38	Iguratimod 25 mg B.i.d+Total Glucosides of Paeony 0.6g T.i.d. + HCQ 0.2g B.i.d	Total Glucosides of Paeony 0.6g T.i.d. + HCQ 0.2g B.i.d	ESR, CRP, Schirmer’s test, Adverse events	57.3 ± 7.92	56.8 ± 8.44	8.8 ± 5.82	9.5 ± 4.86	–	–	24 weeks
Xia et al. 2017(46)	China	50	50	Iguratimod 25 mg B.i.d. + Methylprednisolone	HCQ 200mg B.i.d. + Methylprednisolone	ESR, RF	42. 13 ± 9.97	42.08 ± 9.65	–	–	–	–	12 weeks
Xu et al. 2017(47)	China	47	47	Iguratimod 25 mg B.i.d. + Conventional treatment	Conventional treatment [8 mg methylprednisolone Q.d. +200 mg HCQ B.i.d]	ESSPRI, ESSDAI, ESR, RF, Schirmer’s test	44.5 ± 13.2	45.3 ± 13.1	6.12 ± 1.82	5.96 ± 1.73	6.8 ± 1.7	7.3 ± 1.2	12 weeks
Luo et al. 2018(48)	China	40	40	Iguratimod 25 mg B.i.d. + Conventional treatment	Conventional treatment [8 mg methylprednisolone Q.d. +200 mg HCQ B.i.d]	ESR, RF, adverse events	43.6 ± 10.5	45.2 ± 12.9	0.5-8		–	–	12 weeks
Zhang and Shen(49)	China	43	43	Iguratimod 25 mg B.i.d. + Methylprednisolone 8 mg	8 mg methylprednisolone Q.d. +200 mg HCQ B.i.d	ESSPRI, ESSDAI, ESR, RF, Schirmer’s test, adverse events	40.35 ± 9.41	41.03 ± 10.01	2.31 ± 0.61	2.20 ± 0.52	7.28 ± 1.36	6.98 ± 1.27	12 weeks
Zhang et al. 2019(50)	China	100	100	Iguratimod 25 mg B.i.d. + Conventional treatment	Conventional treatment [Prednisone, HCQ, olfaction]	ESR, adverse events	30.68 ± 3.51	31.00 ± 3.60	1.03 ± 0.22	1.08 ± 0.22	–	–	20 weeks
Liang et al., 2021(51)	China	30	30	Iguratimod 25 mg B.i.d. + Methylprednisolone 8 mg	8 mg methylprednisolone Q.d. +200 mg HCQ B.i.d	ESSDAI, ESSPRI, ESR, CRP, adverse events	45.16 ± 6.37	40.15 ± 6.65	3.59 ± 0.51	3.52 ± 0.50	6.96 ± 0.79	7.12 ± 0.95	16 weeks
Rao et al. 2022(52)	China	43	43	Iguratimod 25 mg B.i.d. + Conventional treatment	Conventional treatment [4 mg methylprednisolone Q.d. +200 mg HCQ B.i.d]	Schirmer’s test, ESR, RF	51.8 ± 10.3	50.1 ± 9.9	2.0 ± 0.5	2.2 ± 0.6	–	–	12 weeks
Chen et al. 2022(39)	China	62	62	Iguratimod 25 mg B.i.d+Total Glucosides of Paeony 0.6g T.i.d. + HCQ 0.2g B.i.d	Total Glucosides of Paeony 0.6g T.i.d. + HCQ 0.2g B.i.d	ESSPRI, ESSDAI, ESR, RF	68. 02 ± 3. 02	68. 50 ± 3. 05	–	–	7. 03 ± 1. 15	7. 04 ± 1. 20	12 weeks
Lu and Zhang(53)	China	48	48	Iguratimod 25 mg B.i.d+ HCQ 0.2g B.i.d	HCQ 0.2g B.i.d	ESR, RF, adverse events	45. 52 ± 7. 48	44. 24 ± 8. 32	3.49 ± 0.26	3.42 ± 0.25	–	–	12 weeks
Jiang et al. 2021(54)	China	24	22	Iguratimod 25 mg B.i.d+ Chere Cunjing Granules	Chere Cunjing Granules (Traditional Chinese Medicine)	ESSPRI, ESSDAI, ESR, CRP, adverse events	45.95 ± 11.52	48.92 ± 11.53	6.36 ± 6.33	4.30 ± 3.44	4.11 ± 1.10	3.93 ± 0.74	12 weeks

### 3.2 Risk of Bias of Included Studies

**Figure 2:**
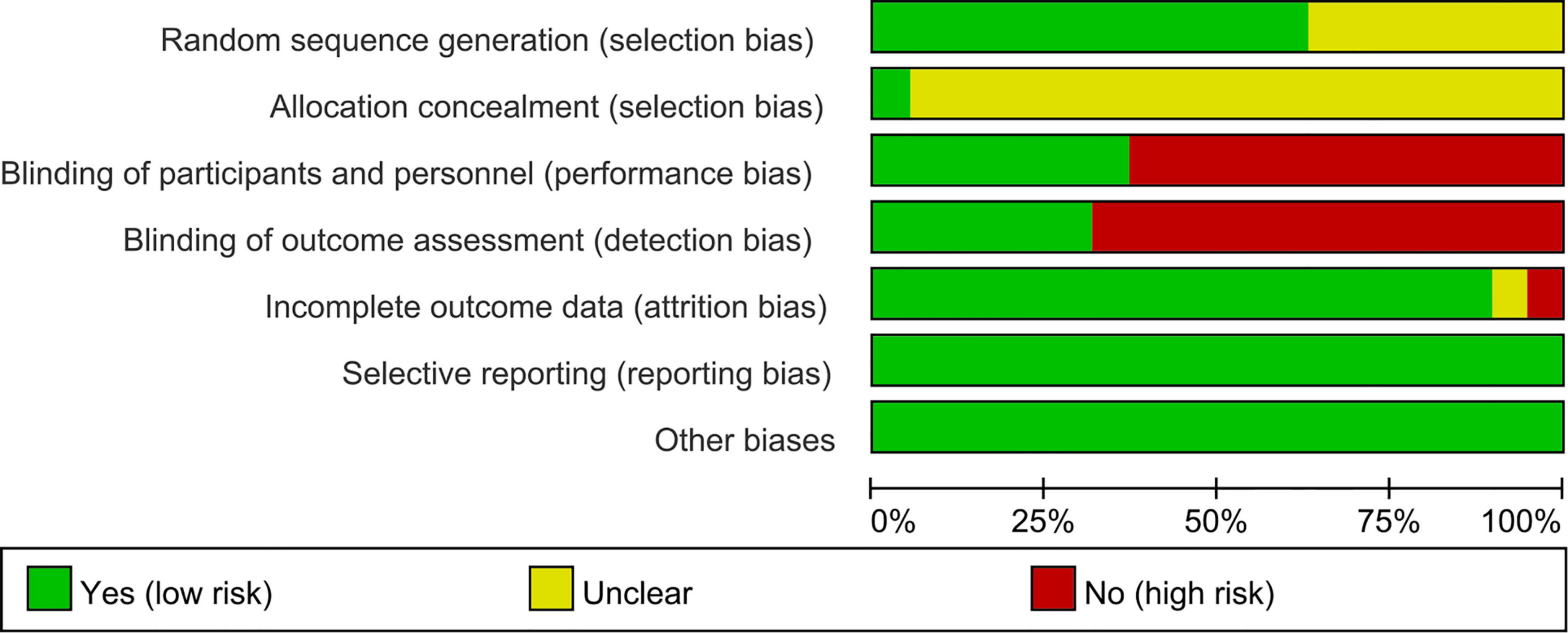
Risk of bias graph.

**Figure 3:**
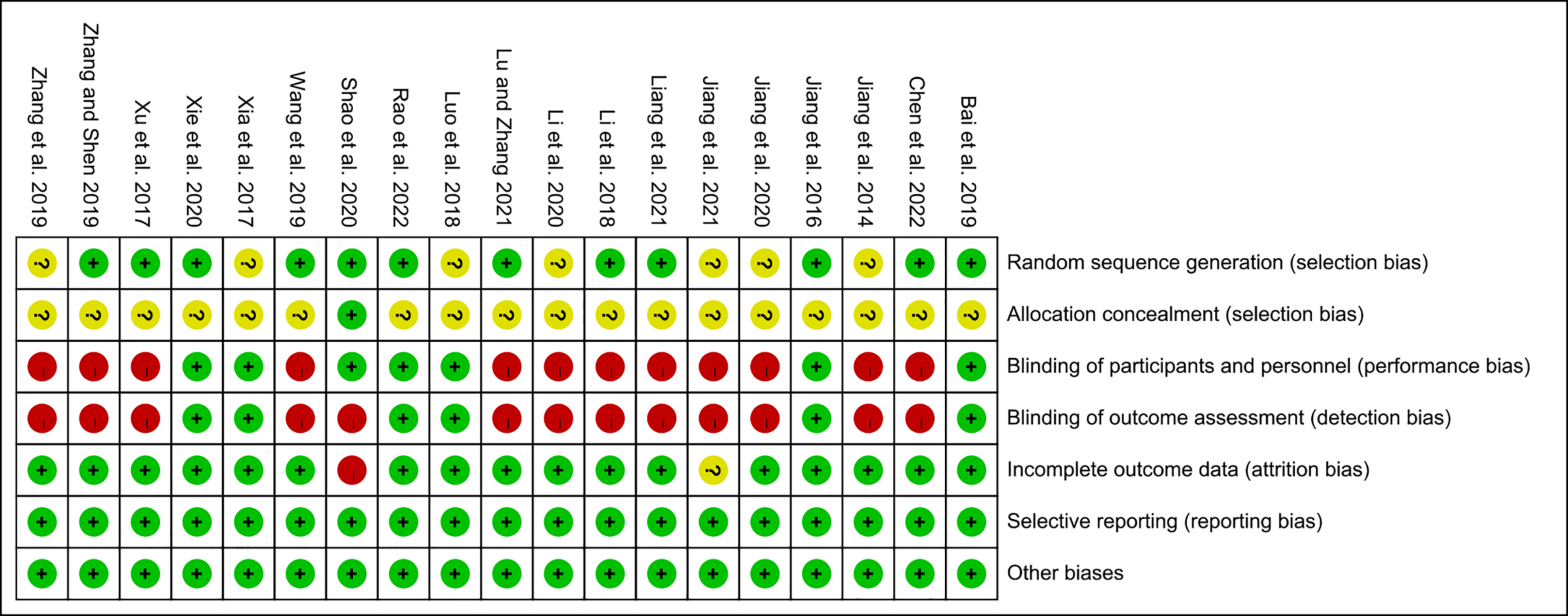
Risk of bias summary.

The summary and graph of risk of bias ware shown in **Figures 2**, **3**.

### 3.3 Primary Outcomes

Primary outcome is Disease activity score (ESSPRI and ESSDAI)

#### 3.3.1 ESSPRI

**Figure 4:**
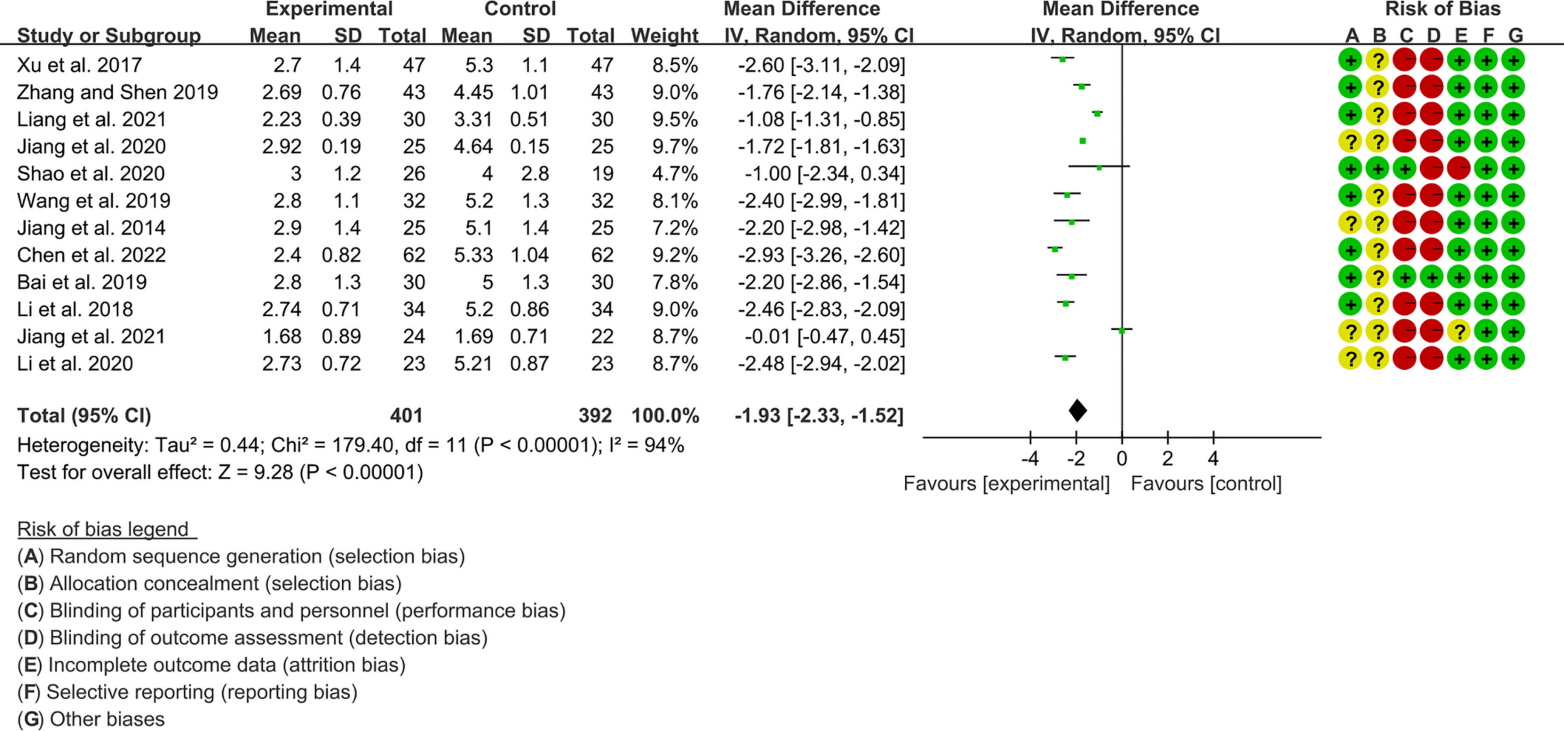
The results of ESSPRI.

Twelve RCTs (401 participants in experimental group and 392 participants in control group) reported ESSPRI. The heterogeneity test P<0.00001, I2 = 94%, indicating that the included studies are highly heterogeneous, and the random effects model was used for analysis. The summary result showed the ESSPRI in experimental group was lower (WMD -1.93 [-2.33, -1.52], P<0.00001; random effect model) (**Figure 4**). The publication bias detection suggests that the possibility of publication bias was small (P=0.514). Referring to MCID, the improvement in ESSPRI was greater than 1, suggesting that the difference between Iguratimod group and the control group is clinically significant.

#### 3.3.2 ESSDAI

**Figure 5:**
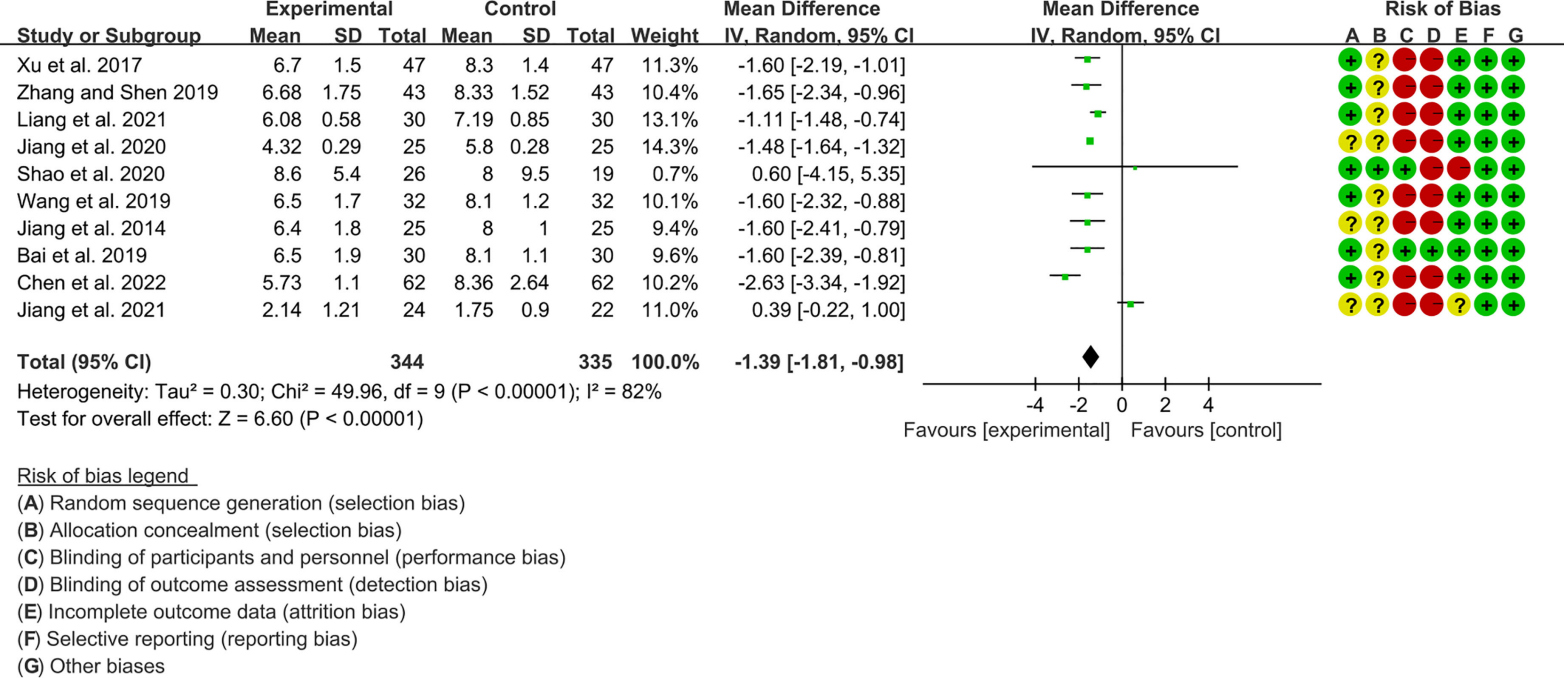
The results of ESSDAI.

Ten RCTs (344 participants in experimental group and 335 participants in control group) reported ESSDAI. The heterogeneity test P<0.00001, I2 = 82%, indicating that the included studies are homogeneous, and the random effects model is used for analysis. The summary result showed the ESSDAI in experimental group was lower (WMD -1.39 [-1.81, -0.98], P<0.00001; random effect model) (**Figure 5**). The publication bias detection suggests that the possibility of publication bias was small (P=0.814). Referring to MCID, the improvement of ESSDAI was less than 3, suggesting that the difference between Iguratimod group and control group is of no clinically significant.

### 3.4 Secondary Outcomes

#### 3.4.1 Schirmer’s Test

**Figure 6:**
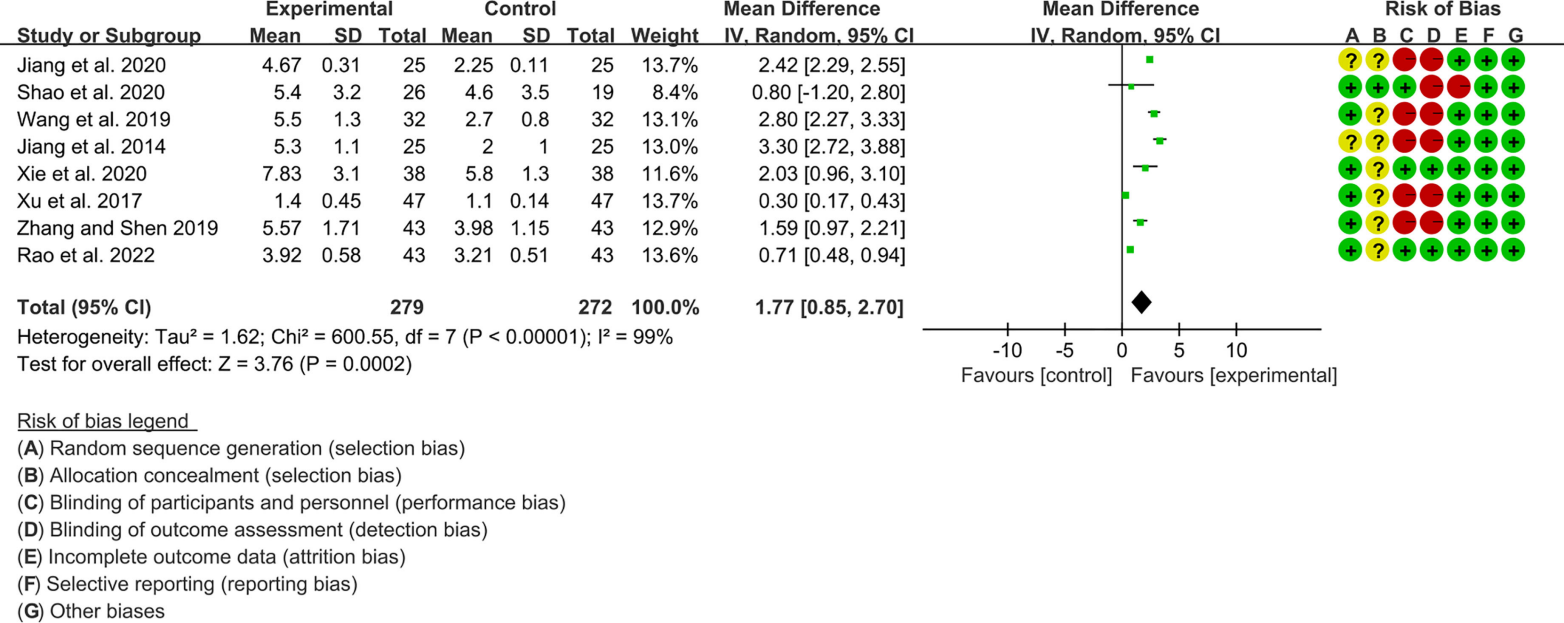
Schirmer’s test.

Eight RCTs, involving 279 participants in experimental group and 272 participants in control group, reported Schirmer’s test. The heterogeneity test P<0.00001, I2 = 99%, indicating that the included studies are highly heterogeneous, and the random effects model was used for analysis. The summary result showed the Schirmer’s test in experimental group was higher (WMD 1.77 [0.85, 2.70], P=0.0002; random effect model) (**Figure 6**). The publication bias detection suggests that the possibility of publication bias was small (P=0.722). Referring to MCID, the improvement of Schirmer’s test was lower than 5 mm, suggesting that the difference between Iguratimod group and control group is of no clinically significant.

#### 3.4.2 The Results of ESR

**Figure 7:**
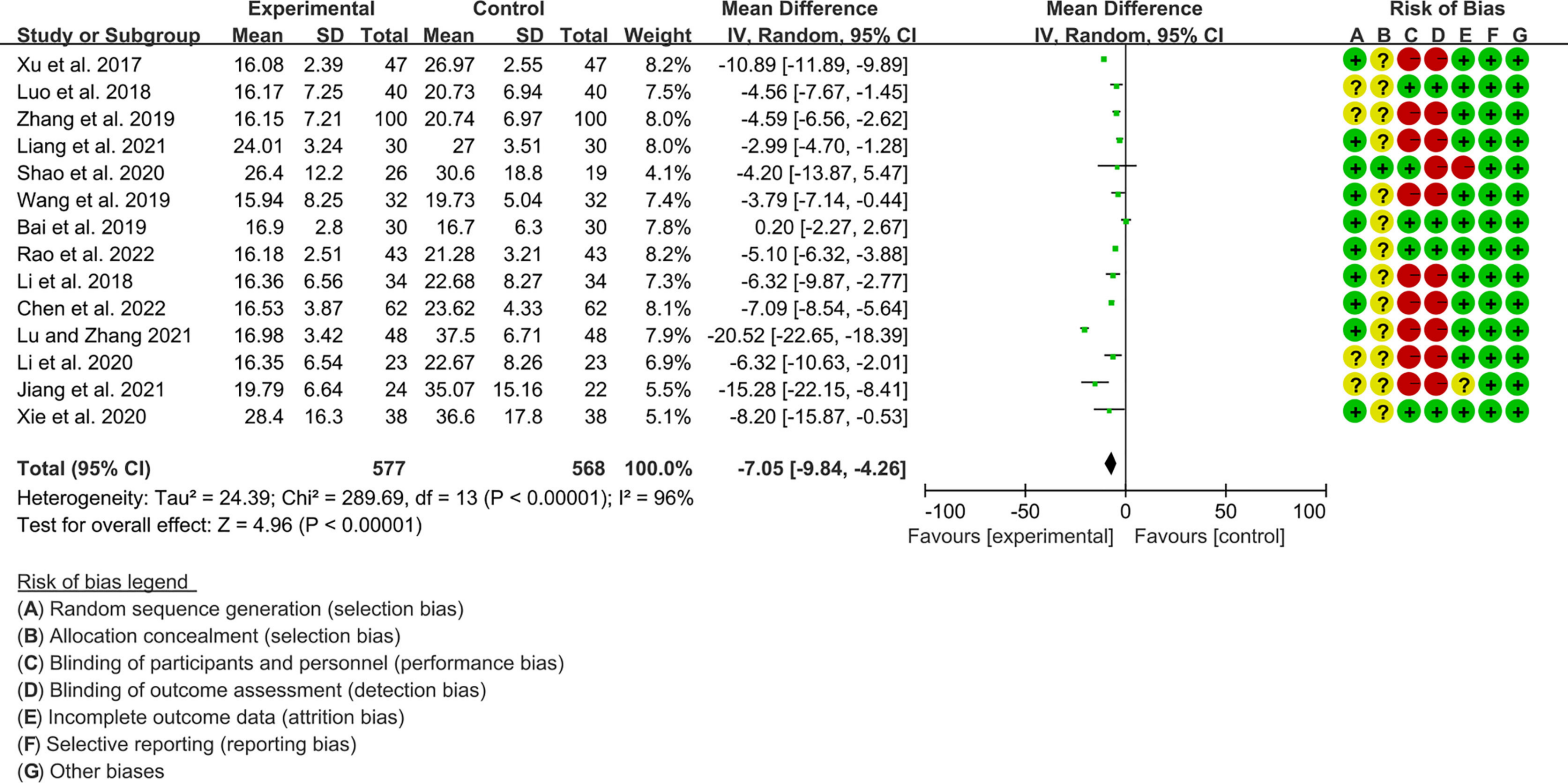
The results of ESR.

Fourteen RCTs (577 participants in experimental group and 568 participants in control group) reported ESR. The heterogeneity test P<0.00001, I2 = 96%, indicating that the included studies are highly heterogeneous, and the random effects model is used for analysis. The summary result showed the ESR in experimental group was lower (WMD -7.05 [-9.84, -4.26], P<0.00001; random effect model) (**Figure 7**).

#### 3.4.3 The Results of CRP

**Figure 8:**
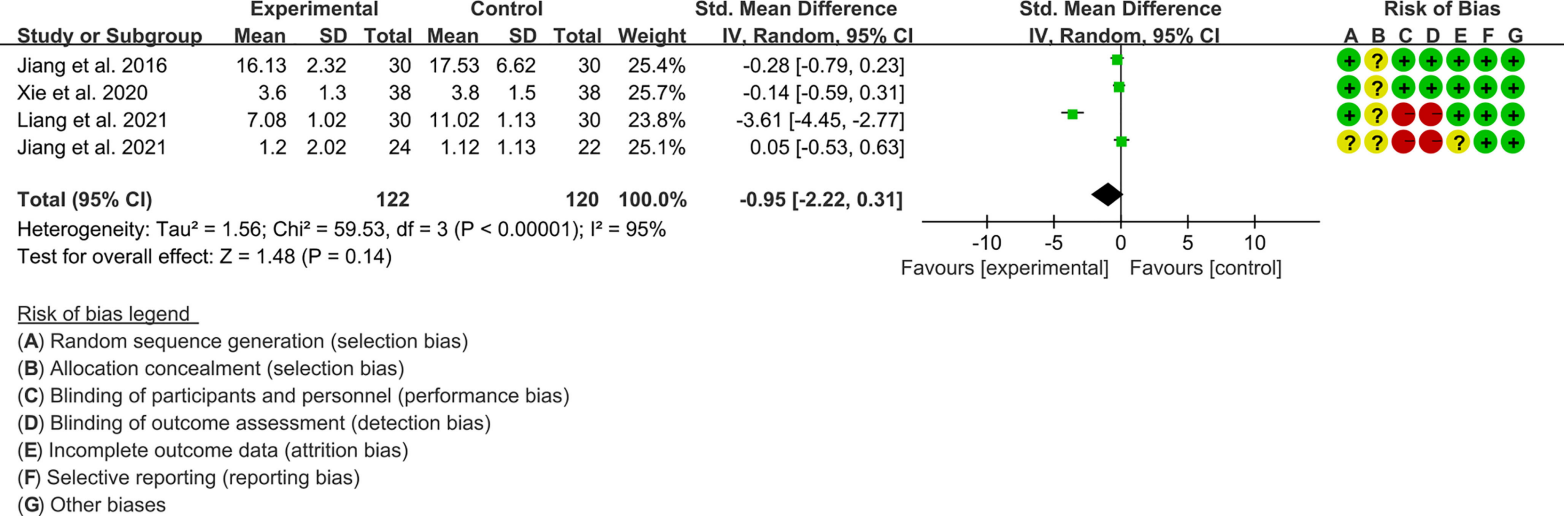
The results of CRP.

Four RCTs (122 participants in experimental group and 120 participants in control group) reported CRP. The heterogeneity test P<0.00001, I2 = 95%, indicating that the included studies are highly heterogeneous, and the random effects model is used for analysis. The summary result showed the CRP between the two groups has no statistical significance (WMD -0.95 [-2.22, 0.31], P=0.14; random effect model) (**Figure 8**).

#### 3.4.4 The Results of RF

**Figure 9:**
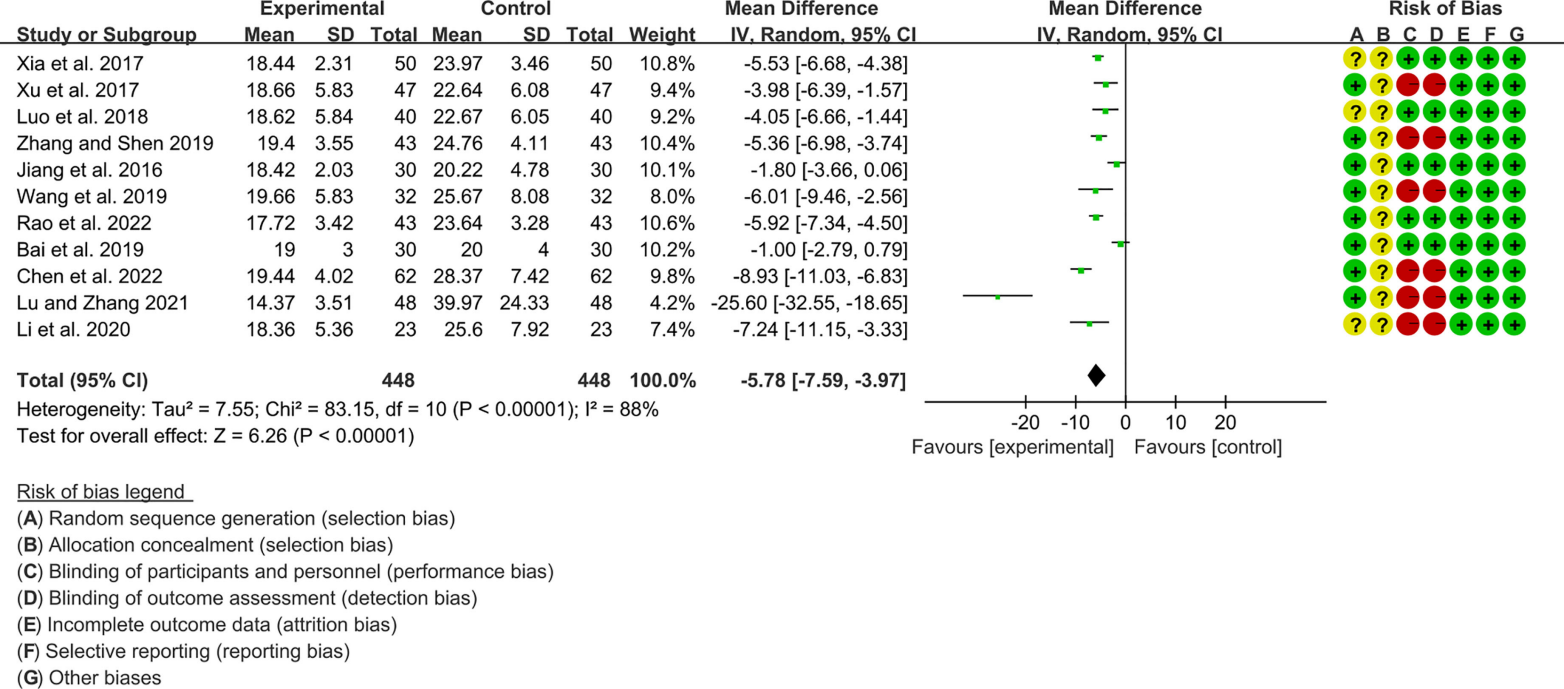
The results of RF.

Eleven RCTs (448 participants in experimental group and 448 participants in control group) reported RF. The heterogeneity test P<0.00001, I2 = 88%, indicating that the included studies are highly heterogeneous, and the random effects model is used for analysis. The summary result showed the RF in experimental group was lower (WMD -5.78 [-7.59, -3.97], P<0.00001; random effect model) (**Figure 9**).

### 3.5 Adverse Events

**Figure 10:**
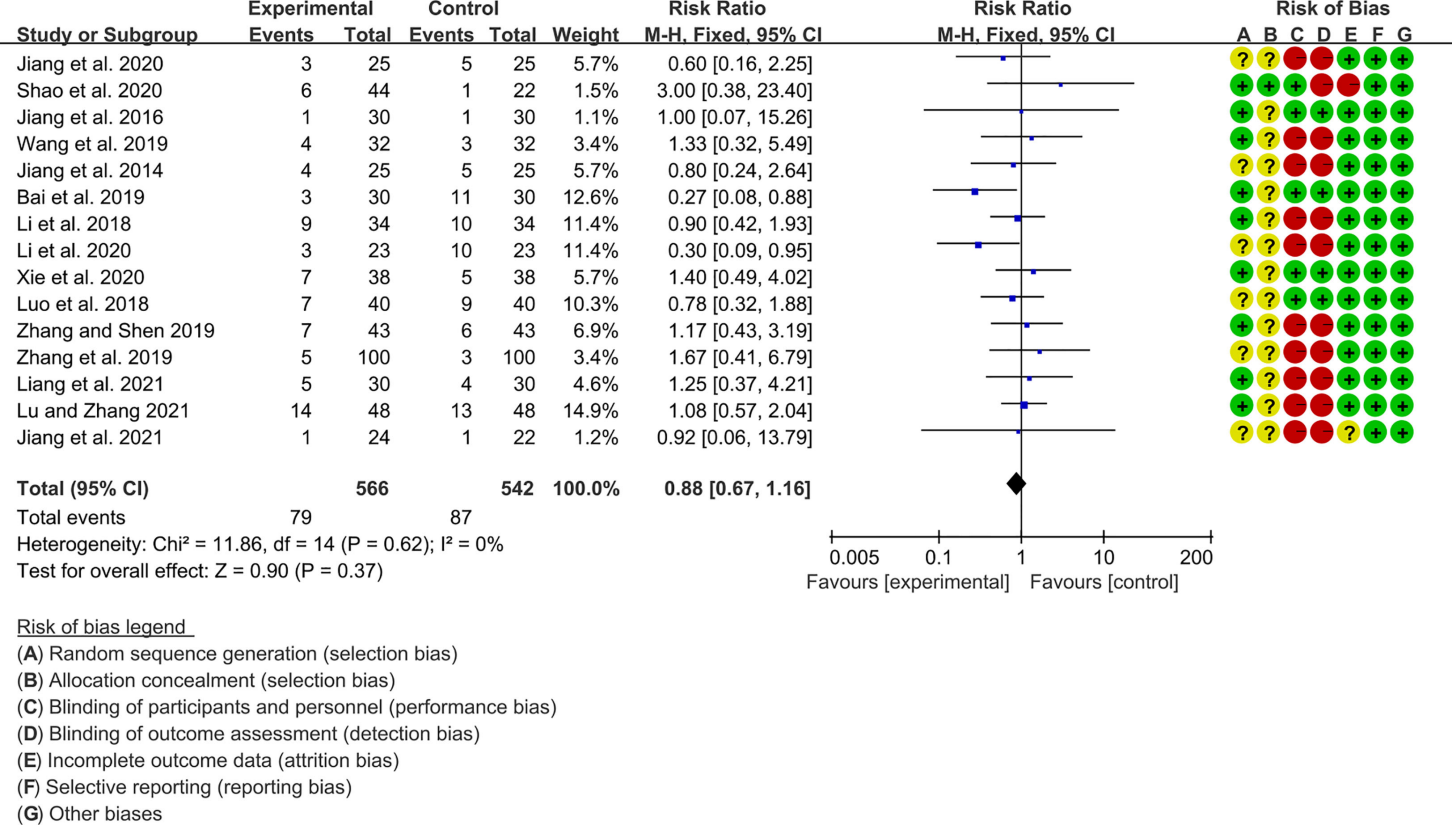
Adverse events.

Fifteen RCTs (566 participants in experimental group and 542 participants in control group) reported adverse events. The heterogeneity test P=0.62, I2 = 0%, indicating that the included studies are heterogeneous, and the fix effects model is used for analysis. The summary result showed the adverse events had a downward trend in the experimental group, but the difference was critical (RR 0.88 [0.67, 1.16], P=0.37; fix effect model) (**Figure 10**). The publication bias detection suggests that the possibility of publication bias was small (P=0.632).

Jiang et al., 2020 (40) showed that 1 case in the iguratimod group and 2 cases in the control group had mildly elevated liver enzyme levels; 2 cases in the iguratimod group and 3 cases in the control group developed diarrhea. Shao et al. 2020 (36) showed 2 cases of elevated liver enzymes in the iguratimod group, 2 cases of gastrointestinal discomfort (1 case in the placebo control group), 1 case of Diarrhoea, and 1 case of Skin rash. Jiang et al. 2016 (41) showed that there was 1 case of liver enzyme elevation and 1 case of leukopenia in the iguratimod group; 1 case of mild abdominal pain and diarrhea, and 1 case of skin pruritus in the control group. (37) showed that there was 1 case of mild liver function abnormality, 1 case of rash with itching, 1 case of diarrhea, and 1 case of leukopenia in the iguratimod group, and 2 cases of diarrhea and 1 case of rash with itching in the control group. Wang et al. 2019 (37) showed that 3 cases of gastrointestinal adverse reactions and 1 case of mild abnormal liver function in the iguratimod group; 2 cases of gastrointestinal discomfort, 2 cases of mild abnormal liver function, and 1 case of suspicious systemic rash in the control group. Bai et al., 2019 (43) showed that 1 case of iguratimod group had abnormal liver function (3 cases in the control group), 1 case with abnormal renal function (2 cases in the control group), and 1 case with abnormal gastrointestinal tract (3 cases in the control group); in addition, there were 3 cases of abnormal blood routine in the control group. Li et al. 2018 (44) showed 2 cases of gastrointestinal discomfort (4 cases in the control group), 1 case of abnormal liver function (1 case in the control group), 1 case of skin rash (2 cases in the control group), 1 case of skin rash (2 cases in the control group), skin itching occurred in 5 cases (3 cases in the control group) in the iguratimod group. Li et al. 2020 (45) showed that 1 case of iguratimod group had gastrointestinal discomfort (4 cases in the control group), 1 case of abnormal liver function (3 cases in the control group), and 1 case of skin rash (3 cases in the control group). Xie et al. 2020 (38) showed that 2 cases of abdominal distension, 2 cases of nausea and vomiting, and 3 cases of diarrhea occurred in the Ailamod group, while 1 case of abdominal distention, 2 cases of nausea and vomiting, 1 case of diarrhea, and 1 case of blurred vision occurred in the control group. Luo et al. 2018 (48) showed that 3 cases of gastrointestinal reactions (2 cases in the control group), 2 cases of rash (2 cases in the control group), 1 case of pruritus (2 cases in the control group), and 1 case of abnormal liver function in the iguratimod group (2 cases in the control group); there was also 1 case of leukopenia in the control group. Zhang and Shen 2019 (49) showed that there were 4 cases of gastrointestinal discomfort, 1 case of rash, 1 case of pruritus, and 1 case of leukopenia in the iguratimod group; in the control group, there were 3 cases of gastrointestinal discomfort, 2 cases of abnormal liver function, and 1 case of skin rash. Zhang et al. 2019 (50) showed that there were 2 cases of gastrointestinal discomfort (2 cases in the control group), 2 cases of abnormal liver function (1 case in the control group) and 1 case of skin rash in the iguratimod group. Liang et al., 2021 (51) showed that 2 cases of gastrointestinal symptoms (1 case in the control group), 2 cases of abnormal liver function (2 cases in the control group) and 1 case of skin itching (1 case in the control group) occurred in the iguratimod group. Lu and Zhang 2021 (53) showed 2 cases of gastrointestinal symptoms (3 cases in the control group), 2 cases of abnormal liver function (2 cases in the control group), 4 cases of skin rash (3 cases in the control group), and 5 cases of skin itching (4 cases in the control group), 1 case of dizziness (1 case in the control group) in the iguratimod group. Jiang et al. 2021 (54) showed that there was 1 case of diarrhea in each of the iguratimod group and the control group. Overall, adverse events were mainly gastrointestinal discomfort, abnormal liver function, and rash and itching.

### 3.6 Subgroup Analysis

Subgroup analyses were performed according to usage of iguratimod, age of patients, duration of intervention, and number of authors of RCTs (**Table 2**).

**Table 2:** Subgroup analysis results.

Subgroup	Outcomes	Overall effect			Heterogeneity test			Statistical method	Studies (N)	Sample size (N)	Figure
		MD	95%CI	P	Tau^2^	I^2^ (%)	P(Q)				
ESSPRI-usage	Once a day	WMD -1.72	[-1.81, -1.63]	P<0.000001	–	–	–	Random	1	50	**Fig S1a**
	Twice a day	WMD -1.94	[-2.51, -1.38]	P<0.000001	0.82	94.37	P<0.000001	Random	11	743
ESSDAI-usage	Once a day	WMD -1.48	[-1.64, -1.32]	P<0.000001	–	–	–	Random	1	50	**Fig S1b**
	Twice a day	WMD -1.38	[-1.95, -0.81]	P<0.00001	0.57	83.32	P<0.000001	Random	9	629
Schirmer’s test-usage	Once a day	WMD 2.42	[2.29, 2.55]	P<0.000001	–	–	–	Random	1	50	**Fig S1c**
	Twice a day	WMD 1.67	[0.85, 2.50]	P=0.00007	1.07	96.75	P<0.000001	Random	7	501
CRP-usage	Once a day	SMD -0.28	[-0.79, 0.23]	P=0.28	–	–	–	Random	1	60	**Fig S1d**
	Twice a day	SMD -1.20	[-3.08, 0.67]	P=0.21	2.64	96.57	P<0.000001	Random	3	182
RF-usage	Once a day	WMD -1.80	[-3.66, 0.06]	P=0.058	–	–	–	Random	1	60	**Fig S1e**
	Twice a day	WMD -6.21	[-8.08, -4.34]	P<0.000001	7.20	87.21	P<0.000001	Random	10	836
Adverse events-usage	Once a day	RR 0.90	[0.67, 1.19]	P=0.45	–	0	P=0.49	Fixed	13	998	**Fig S1f**
	Twice a day	RR 0.67	[0.20, 2.17]	P=0.50	–	0	P=0.74	Fixed	2	110
ESSPRI-age	> 60 years old	WMD -2.72	[-3.23, -2.22]	P<0.000001	0.08	57.67	P=0.12	Random	2	188	**Fig S2a**
	<60 years old	WMD -1.77	[-2.18, -1.36]	P<0.000001	0.36	92.67	P<0.000001	Random	10	605
ESSDAI-age	> 60 years old	WMD -2.12	[-3.13, -1.11]	P<0.0001	0.40	74.81	P=0.046	Random	2	188	**Fig S2b**
	<60 years old	WMD -1.21	[-1.65, -0.77]	P<0.000001	0.27	81.56	P<0.00001	Random	8	491
Schirmer’s test-age	> 60 years old	WMD 2.80	[2.27, 3.33]	P<0.000001	–	–	–	Random	1	64	**Fig S2c**
	< 60 years old	WMD 1.62	[0.63, 2.61]	P=0.001	1.62	98.95	P<0.000001	Random	7	487
ESR-age	> 60 years old	WMD -5.80	[-8.96, -2.64]	P=0.0003	3.71	68.18	P=0.08	Random	2	188	**Fig S2d**
	< 60 years old	WMD -7.34	[-10.67, -4.01]	P=0.00002	30.14	96.13	P<0.000001	Random	12	957
RF-age	> 60 years old	WMD -7.80	[-10.59, -5.02]	P<0.000001	2.14	50.14	P=0.16	Random	2	188	**Fig S2e**
	< 60 years old	WMD -5.36	[-7.31, -3.40]	P<0.000001	7.17	88.26	P<0.000001	Random	9	708
Adverse events-age	< 60 years old	RR 0.86	[0.65, 1.15]	P=0.31	–	0	P=0.57	Fixed	14	1044	**Fig S2f**
	> 60 years old	RR 1.33	[0.32, 5.49]	P=0.69	–	–	–	Fixed	1	64
ESSPRI-duration	12 weeks	WMD -2.07	[-2.52, -1.62]	P<0.000001	0.47	93.57	P<0.000001	Random	10	688	**Fig S3a**
	16 weeks	WMD -1.08	[-1.31, -0.85]	P<0.000001	–	–	–	Random	1	60
	24 weeks	WMD -1.00	[-2.34, 0.34]	P=0.14	–	–	–	Random	1	45
ESSDAI-duration	12 weeks	WMD -1.46	[-1.96, -0.96]	P<0.000001	0.41	84.88	P<0.000001	Random	8	574	**Fig S3b**
	16 weeks	WMD -1.11	[-1.48, -0.74]	P<0.000001	–	–	–	Random	1	60
	24 weeks	WMD 0.60	[-4.15, 5.35]	P=0.8	–	–	–	Random	1	45
Schirmer’s test-duration	12 weeks	WMD 1.84	[0.80, 2.88]	P=0.0005	1.64	99.17	P<0.000001	Random	6	430	**Fig S3c**
	24 weeks	WMD 1.72	[0.67, 2.77]	P=0.0014	0.09	11.70	P=0.29	Random	2	121
ESR-duration	12 weeks	WMD -7.84	[-11.22, -4.45]	P<0.000001	27.16	96.33	P<0.000001	Random	10	764	**Fig S3d**
	12-24 weeks	WMD -3.71	[-5.27, -2.15]	P<0.000001	0.40	31.01	P=0.23	Random	2	260
	24 weeks	WMD -6.65	[-12.66, -0.64]	P=0.03	0.00	0.00	P=0.53	Random	2	121
CRP-duration	12 weeks	SMD -0.14	[-0.52, 0.25]	P=0.48	0.00	0.00	P=0.41	Random	2	106	**Fig S3e**
	16 weeks	SMD -3.61	[-4.45, -2.77]	P<0.000001	–	–	–	Random	1	60
	24 weeks	SMD -0.14	[-0.59, 0.31]	P=0.54	–	–	–	Random	1	76
Adverse events-duration	12 weeks	RR 0.76	[0.55, 1.03]	P=0.08	–	0	P=0.60	Fixed	11	706	**Fig S3f**
	12-24 weeks	RR 1.43	[0.57, 3.58]	P=0.45	–	0	P=0.76	Fixed	2	260
	24 weeks	RR 1.74	[0.68, 4.45]	P=0.25	–	0	P=0.51	Fixed	2	142
ESSPRI-number of authors	> 3 authors	WMD -1.70	[-2.20, -1.21]	P<0.000001	0.42	93.87	P<0.000001	Random	8	477	**Fig S4a**
	≤ 3 authors	WMD -2.35	[-2.93, -1.77]	P<0.000001	0.29	85.99	P<0.0001	Random	4	316
ESSDAI-number of authors	> 3 authors	WMD -1.15	[-1.64, -0.65]	P<0.00001	0.30	83.94	P<0.00001	Random	7	409	**Fig S4b**
	≤ 3 authors	WMD -1.97	[-2.63, -1.30]	P<0.000001	0.21	59.78	P=0.08	Random	3	270
Schirmer’s test-number of authors	> 3 authors	WMD 1.80	[0.79, 2.81]	P=0.0005	1.68	99.00	P<0.000001	Random	7	465	**Fig S4c**
	≤ 3 authors	WMD 1.59	[0.97, 2.21]	P<0.000001	–	–	–	Random	1	86
ESR-number of authors	> 3 authors	WMD -6.28	[-8.85, -3.72]	P<0.00001	12.97	91.55	P<0.000001	Random	10	819	**Fig S4d**
	≤ 3 authors	WMD -8.46	[-17.15, 0.22]	P=0.056	76.47	98.28	P<0.000001	Random	4	326
RF-number of authors	> 3 authors	WMD -4.56	[-5.93, -3.18]	P<0.000001	1.82	67.05	P=0.01	Random	6	484	**Fig S4e**
	≤ 3 authors	WMD -8.53	[-13.04, -4.02]	P=0.0002	23.28	94.06	P<0.000001	Random	5	412
Adverse events-number of authors	> 3 authors	RR 1.05	[0.73, 1.52]	P=0.78	–	0	P=0.97	Fixed	11	820	**Fig S4f**
	≤ 3 authors	RR 0.68	[0.44, 1.04]	P=0.08	–	59.35	P=0.06	Fixed	4	288

#### 3.6.1 Usage of Iguratimod

The included RCTs showed the use of iguratimod once or twice a day. Subgroup analysis showed that iguratimod 50 mg once a day and iguratimod 25 mg twice a day were both positive in Adverse events, CRP, ESSDAI, ESSPRI, and Schirmer’s test. However, the results of iguratimod once a day were negative, which was inconsistent with the results of iguratimod twice a day. Since there are too few RCTs in the “once a day” subgroup, more RCTs are needed to further test the results. Referring to MCID, the improvement of ESSPRI is clinically significant, while the improvement of ESSDAI and Schirmer’s test is of no clinically significant.

#### 3.6.2 Age of Patients

RCTs were divided into “>60 years subgroup” and “<60 years old subgroup” according to the age of enrolled patients. Subgroup analyses showed that iguratimod was both effective and safe for people younger or older than 60. Referring to MCID, the improvement of ESSPRI is clinically significant, while the improvement of Schirmer’s test is of no clinically significant. Meanwhile, the improvement of ESSDAI in “> 60 years old” subgroup may be clinically significant.

#### 3.6.3 Duration of Intervention

RCTs were divided into 12 weeks, 12-24weeks and 24weeks according to the duration of intervention. Strangely, the results for the “24 weeks” subgroup of ESSDAI and ESSPRI were negative. Since only 1 RCT is involved, its reliability is not high. More long-term RCTs are needed for further exploration. Referring to MCID, the improvement of ESSPRI in “12 weeks” subgroup is clinically significant, while the improvement of ESSDAI and Schirmer’s test is of no clinically significant.

#### 3.6.4 Number of Authors of RCTs

Subgroup analysis was performed according to the authors greater than 3 or less than or equal to 3 to test the consistency of the results of the RCTs. The results of subgroup analysis showed that the results of the “>3 authors” subgroup and the “≤3 authors” subgroup were basically the same. Only ESR showed inconsistent results, which may be due to individual differences. Referring to MCID, the improvement of ESSPRI is clinically significant, while the improvement of ESSDAI and Schirmer’s test is of no clinically significant.

### 3.7 Sensitivity Analysis

**Figure 11:**
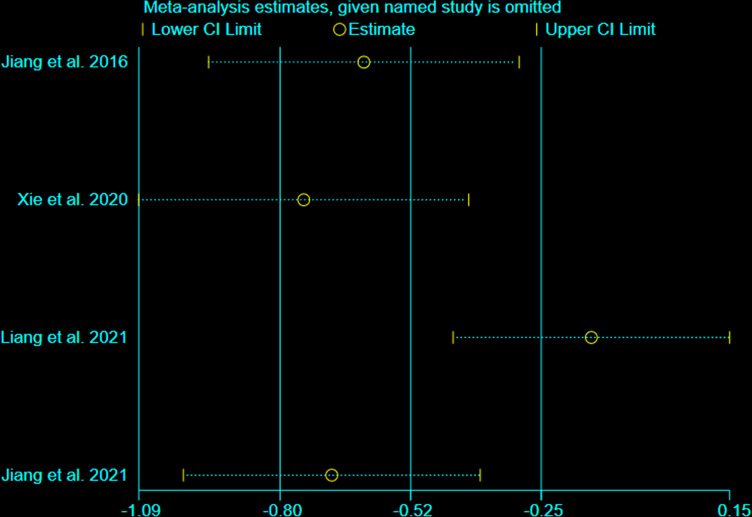
sensitivity analysis of CRP.

We undertook sensitivity analysis for CRP (**Figure 11**). After we omitted the Liang et al., 2021, we found that the results changed significantly. This suggests that this outcome is not stable.

## 4 Discussion

### 4.1 Main Findings

This systematic review and meta-analysis included 19 RCTs with 1512 participants. The statistical analysis results showed that Iguratimod can reduce the ESSPRI score and ESSDAI score, and increase Schirmer’s test score; and the improvement of ESSPRI score is clinically significant. Iguratimod can also reduce systemic inflammation (decrease ESR level) and decrease RF. For CRP, this research showed that there was no statistical significance between experimental group and control group. Meanwhile, the addition of Iguratimod may not increase the occurrence of adverse events. The heterogeneity test found that the results of ESSPRI, ESSDAI, Schirmer’s test, ESR, CRP, RF are highly heterogeneous. After careful reading of the included RCTs, it is found that the interventions of these RCTs are not exactly the same, which may cause one of the sources of heterogeneity. For the results of CRP, the results of the fixed effect model are inconsistent with that of the random effect model; hence, the sensitivity analysis was performed on them. after we omitted the Liang et al., 2021, we found that the results changed significantly. This suggests that this outcome is not stable. We also used the Egger method and Harbord method to detect the publication bias of the main results, and showed that both P>0.1, indicating that there is no publication bias in the study. In addition, we performed subgroup analysis according to usage of iguratimod, age of patients, duration of intervention, and number of authors of RCTs. We found that the results of the subgroup analysis were basically consistent with the results of the pooled analysis. Although there are inconsistent results (for example, the results for the “24 weeks” subgroup of ESSDAI and ESSPRI were negative), it only contains 1 RCT, the reliability of the results is not high enough, and more RCTs are needed to confirm or correct the results. Interestingly, although the improvement in summary ESSDAI was not clinically significant, subgroup analysis showed that the results in the “>60 years” subgroup may be clinically significant. This suggests that iguratimod may have potential benefits for people over the age of 60.

### 4.2 Interpretation of the Outcomes

As a systemic autoimmune disease, pSS currently has a heavy burden of disease research, and there is currently no specific treatment for pSS. The current comprehensive clinical management mainly includes local treatment and systemic treatment according to the patient’s symptoms. For xerostomia, muscarinic agonists and artificial salivary replacement therapy are preferred without contraindications. For severe exocrine gland inflammation or involvement of extraglandular systems (such as skin, joints, muscles), systemic therapy (glucocorticoids, immunosuppressants, non-steroidal anti-inflammatory drugs, biological agents, etc.) is used. Although the above traditional anti-rheumatic drugs to improve the condition of the disease are supported by expert opinions, non-controlled studies and daily clinical practice, the effectiveness and safety of these anti-rheumatic drugs in the treatment of pSS still have not been verified by large-scale RCTs. According to the recommendations provided by the pSS diagnosis and treatment guidelines, hydroxychloroquine is the first-line drug for the treatment of inflammatory manifestations of the musculoskeletal system. A small dose/short course of glucocorticoids can be used for parotid gland enlargement, arthritis and skin vasculitis. Cyclophosphamide and azathioprine are effective drugs for the treatment of severe vasculitis or central nervous system involvement. Rituximab is used to treat pSS-related lymphoma. However, due to the many adverse reactions of non-steroidal anti-inflammatory drugs, glucocorticoids and the high price of biological preparations, there are high hopes for exploring new anti-rheumatic drugs to improve the disease. Current research shows that Iguratimod, as a new type of small molecule anti-rheumatic drug, has shown clinical effectiveness in the treatment of autoimmune diseases (such as rheumatoid arthritis and pSS). Its pharmacological mechanism is mainly related to inhibit the production of immunoglobulin and autoantibodies by B cells, down-regulate T cell-mediated cellular immunity, and inhibit various inflammatory factors (IL-1, IL-6, IL-8 and TNF) and the expression of inflammatory signal pathway (NF-KB signal pathway) [Jiang et al., 2020 (55)]. This research showed that Iguratimod combined with DMARDs can reduce the ESSPRI score, ESSDAI score, RF, and systemic inflammation (decrease the IgG and IgA level and ESR), and increase Schirmer’s test score, Saliva flow rate and PLT. Our previous open-label pilot study also showed that Iguratimod can reduce ESSDAI, RF and IgG level (56).

The results of this meta-analysis showed that the incidence of adverse reactions in the iguratimod test group was lower than that in the control group (P<0.05), indicating that iguratimod did not increase the adverse reaction rate and had better safety. Several meta-analysis of systematic reviews of iguratimod intervention in rheumatoid arthritis showed that Iguratimod is safe (57, 58). In addition, a number of clinical studies over 52 weeks have proved that Iguratimod can maintain good efficacy and safety regardless of long-term use as a single drug or a combination (59–64).

### 4.3 Quality of the Evidence

The GRADE tool was utilized to rate the quality of the evidence (65). According to the GRADE handbook (66), the evidence was judged to be high to low. The risk of bias associated with the unclear random sequence generation, allocation concealment, blinding and incomplete outcomes. Meanwhile, some of the evidence was downgraded because of their inconsistency and/or imprecision. The details were described in **Table 3**.

**Table 3:** Summary of findings for the main comparison.

**Iguratimod intervention in pSS**						
**Patient or population: patients with pSS Intervention: Iguratimod or combined with iguratimod on the basis of the control group Comparison: placebo treatment or conventional therapy**						
**Outcomes**	Illustrative comparative risks* (95% CI)		**Relative effect** **(95% CI)**	**No of Participants** **(studies)**	**Quality of the evidence** **(GRADE)**	**Comments**
	**Assumed risk**	**Corresponding risk**				
	**Control**	**Primary outcomes**				
**Adverse events**	**Study population**		**RR 0.88** (0.67 to 1.16)	1108 (15 studies)	⊕⊕⊕⊝ **moderate**^1^	
	**161 per 1000**	**141 per 1000** (108 to 186)		
	**Moderate**			
	**140 per 1000**	**123 per 1000** (94 to 162)		
**ESSPRI**		The mean esspri in the intervention groups was **1.93 lower** (2.33 to 1.52 lower)		793 (12 studies)	⊕⊕⊝⊝ **low**^1,2^	
**ESSDAI**		The mean essdai in the intervention groups was **1.39 lower** (1.81 to 0.98 lower)		679 (10 studies)	⊕⊕⊝⊝ **low**^1,2^	
**Schirmer’s test**		The mean schirmer’s test in the intervention groups was **1.77 higher** (0.85 to 2.7 higher)		551 (8 studies)	⊕⊕⊝⊝ **low**^1,2^	

*The basis for the assumed risk (e.g. the median control group risk across studies) is provided in footnotes. The corresponding risk (and its 95% confidence interval) is based on the assumed risk in the comparison group and the relative effect of the intervention (and its 95% CI).

CI, Confidence interval; RR, Risk ratio;

GRADE Working Group grades of evidence.

High quality: Further research is very unlikely to change our confidence in the estimate of effect.

Moderate quality: Further research is likely to have an important impact on our confidence in the estimate of effect and may change the estimate.

Low quality: Further research is very likely to have an important impact on our confidence in the estimate of effect and is likely to change the estimate.

Very low quality: We are very uncertain about the estimate.

^1^Downgraded one level due to serious risk of bias (random sequence generation, allocation concealment, blinding, incomplete outcomes) and most of the data comes from the RCTs with moderate risk of bias.

^2^Downgraded one level due to the probably substantial heterogeneity.

### 4.4 The Strengths of This Review

This is a comprehensive and newest systematic review and meta-analysis of RCTs on iguratimod intervention in pSS. This research not only found that the addition of iguratimod can improve the symptoms of pSS patients, but also showed that it does not increase adverse events. This research also conducted a quality assessment of primary outcomes to provide references for clinical applications and scientific research. Compared with previous systematic review and meta-analysis (21), the strengths of this review are: (1) Stricter inclusion and exclusion criteria were applied, excluding articles with only 1 author (even if they claimed to be an RCT). And the number of authors was used as a subgroup analysis to determine the consistency of the results of RCTs, and found that the results were consistent. (2) We introduced MCID as an assessment criterion for clinical efficacy and found that the improvement of ESSPRI is clinically significant, while the improvement of ESSDAI and Schirmer’s test is of no clinically significant. (3) In addition, subgroup analysis was performed according to usage of iguratimod, age of patients, duration of intervention. And it was found that iguratimod is effective whether 50mg Q.d or 25mg B.i.d; iguratimod is effective in people of all ages, but the improvement in ESSDAI may be more clinically significant in people older than 60 years than in people younger than 60 years. And based on the current evidence, it is speculated that the iguratimod intervention needs to continue for at least 12 weeks.

### 4.5 The Limitations of This Review

(1) The research area of all RCTs is in China and the participants are also Chinese. There is a lack of data from other countries and races. (2) Although there were 19 RCTs, the number of participants was small (The total number of participants does not exceed 4,000, and the number of participants in a single RCT does not exceed 150); some subgroups even had only 1 RCT. (3) Most RCTs failed to perform allocation concealment and blinding, which leads to a high risk of bias. (4) The quality of primary outcomes was also low; those outcomes was downgraded because of the serious risk of bias, the total sample size fails to meet the optimal information size. (5) For the subgroup analysis of age, due to the different age ranges involved in different RCTs, the subgroups can only be roughly divided into <60 years old and >60 years old, but cannot be divided into 30-40, 40-50,… and other levels. (6) Most of the RCTs were treated for 12 weeks, and there is a lack of observations on the efficacy of shorter (such as 4 weeks, 8 weeks) or longer treatment periods (48 weeks and more). This makes it difficult to evaluate the course of treatment finely.

### 4.6 Implications for Future Clinical Practice and Future Study

This systematic review and meta-analysis provides evidence of the use of the iguratimod in clinical practice. The results of this study support the application of iguratimod to pSS patients, because iguratimod can improve patients’ clinical symptoms (such as ESSPRI score, ESSDAI score, Schirmer’s test score) and systemic inflammatory response (decrease ESR level) and has good safety. From this point of view, iguratimod can be combined with a variety of DMARDs in the treatment of pSS, such as HCQ, and the combination therapy can have both efficacy and safety. However, further RCTs coming from different countries and races are needed to explore the efficacy and safety of iguratimod for other countries and races. More high-quality RCTs using blinding and allocation concealment are also demanded to reduce the risk of bias and generate more reliable and higher-quality evidence. In view of the small number of participants in the RCTs in this meta-analysis, more large-sample, multi-center RCT also expected so as to the findings of this research. In the future, more studies with courses of 4, 8, and 48 weeks, as well as more refined age-stratified studies, are needed to evaluate the efficacy and safety of IGU for different intervention times and for different age groups. More basic research about the iguratimod intervention in pSS are needed to explore the mechanisms of this medicine.

## 5 Conclusion

Based on current evidence, iguratimod can effectively reduce ESSPRI score, ESSDAI score, Schirmer’s test score and decrease systemic inflammatory response (such as ESR level and RF level) without increasing the probability of adverse events. And its improvement of ESSPRI has clinical significance, and the improvement of ESSDAI in people > 60 years old may have clinical significance. The recommended course of treatment is at least 12 weeks.

## Data Availability Statement

The original contributions presented in the study are included in the article/**Supplementary Material**. Further inquiries can be directed to the corresponding authors.

## Author Contributions

LZ, GY, HC are responsible for the study concept and design. LZ, KY, WH, GY, QH and HC are responsible for the data collection, data analysis and interpretation; LZ and KY drafted the paper;HC and GY supervised the study; all authors participated in the analysis and interpretation of data and approved the final paper.

## Funding

This work is supported by Natural Science Foundation of Changsha City (No. kq2202500).

## Conflict of Interest

The authors declare that the research was conducted in the absence of any commercial or financial relationships that could be construed as a potential conflict of interest.

## Publisher’s Note

All claims expressed in this article are solely those of the authors and do not necessarily represent those of their affiliated organizations, or those of the publisher, the editors and the reviewers. Any product that may be evaluated in this article, or claim that may be made by its manufacturer, is not guaranteed or endorsed by the publisher.
