# Supplementary figures and images for: A Systematic Review and Meta-Analysis of 19 Randomized Controlled Trials of Iguratimod Combined With Other Therapies for Sjogren’s Syndrome
Source: Front Immunol. 2022 Jul 28;13:924730. doi: 10.3389/fimmu.2022.924730 (PMC9367640; doi:10.3389/fimmu.2022.924730)

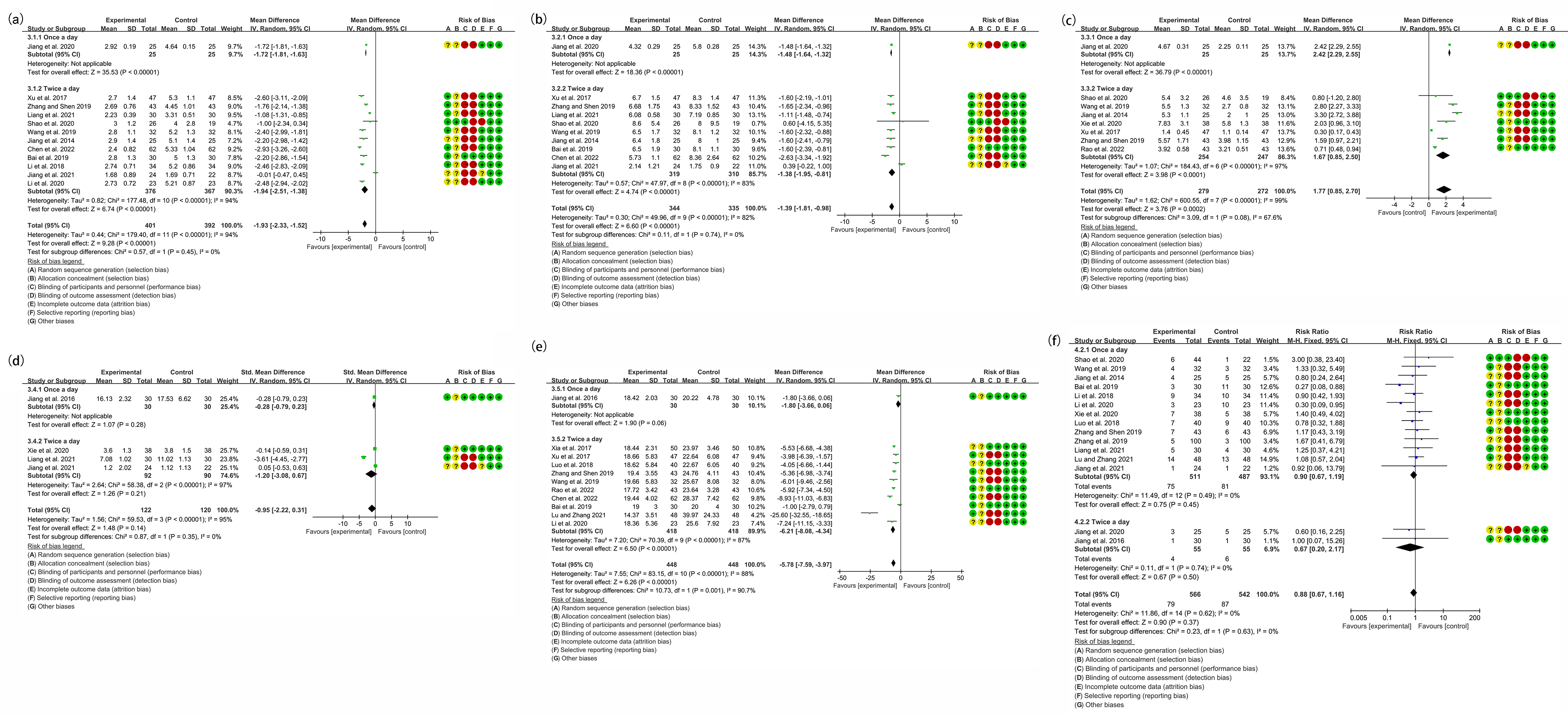

Supplement: Supplementary file 1 [file Image_1.tif]

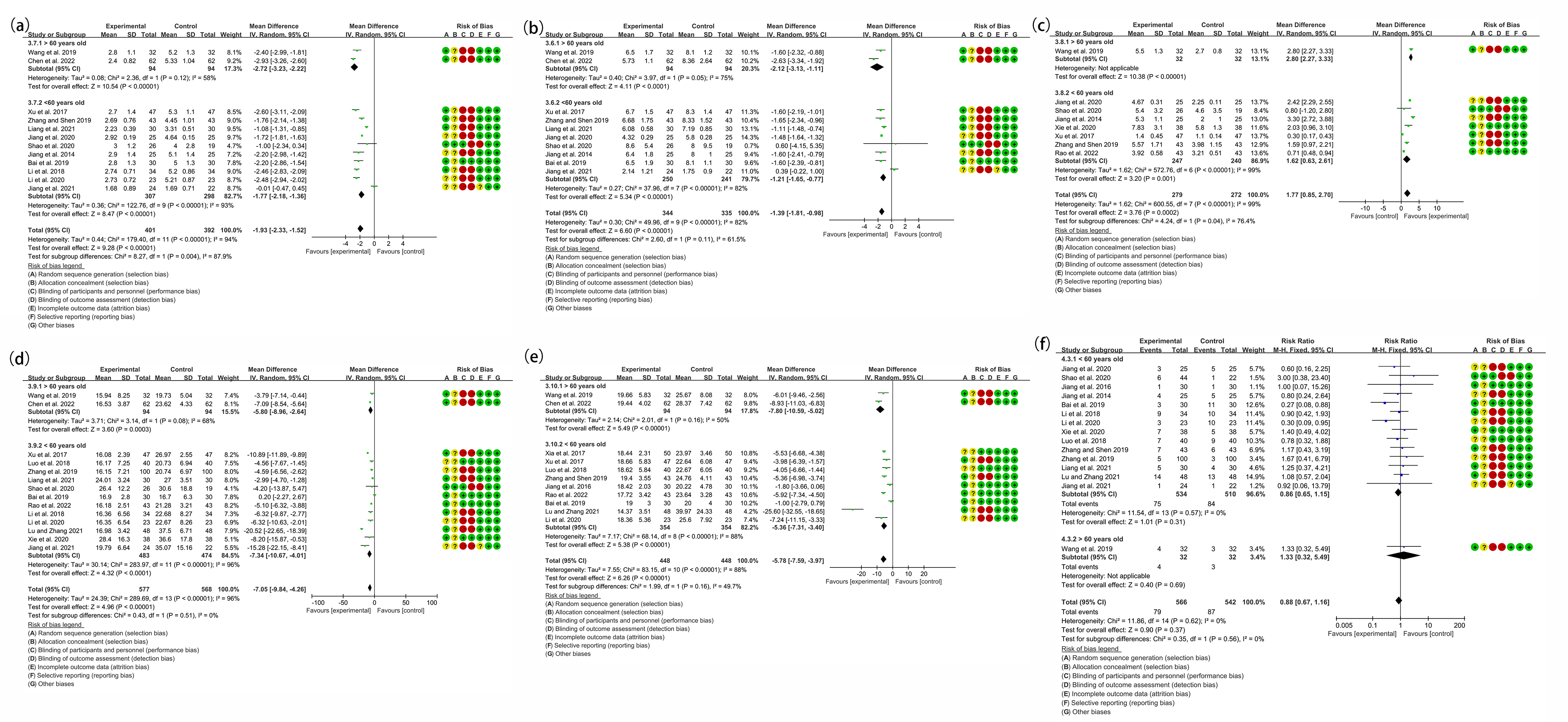

Supplement: Supplementary file 2 [file Image_2.tif]

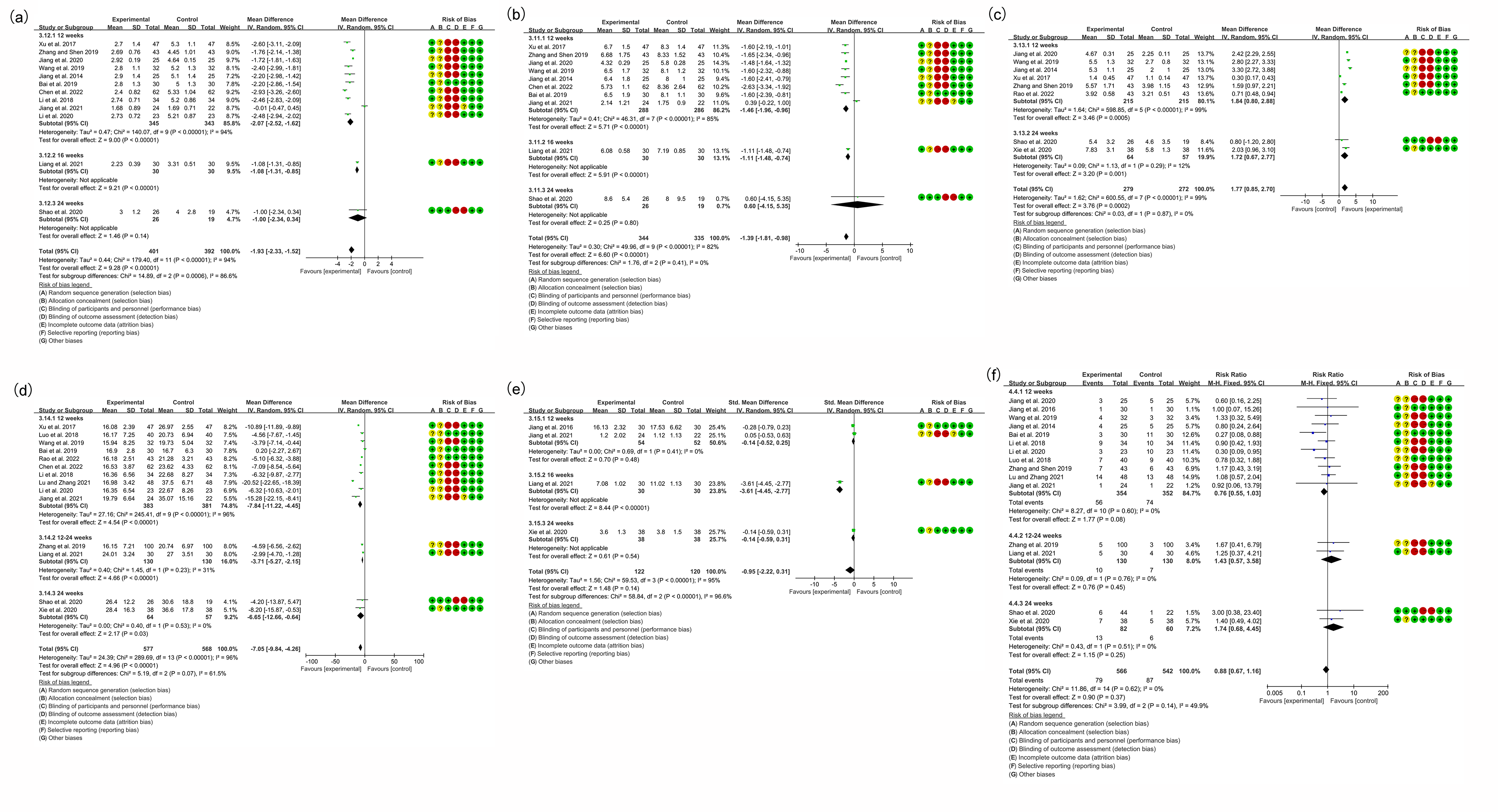

Supplement: Supplementary file 3 [file Image_3.tif]

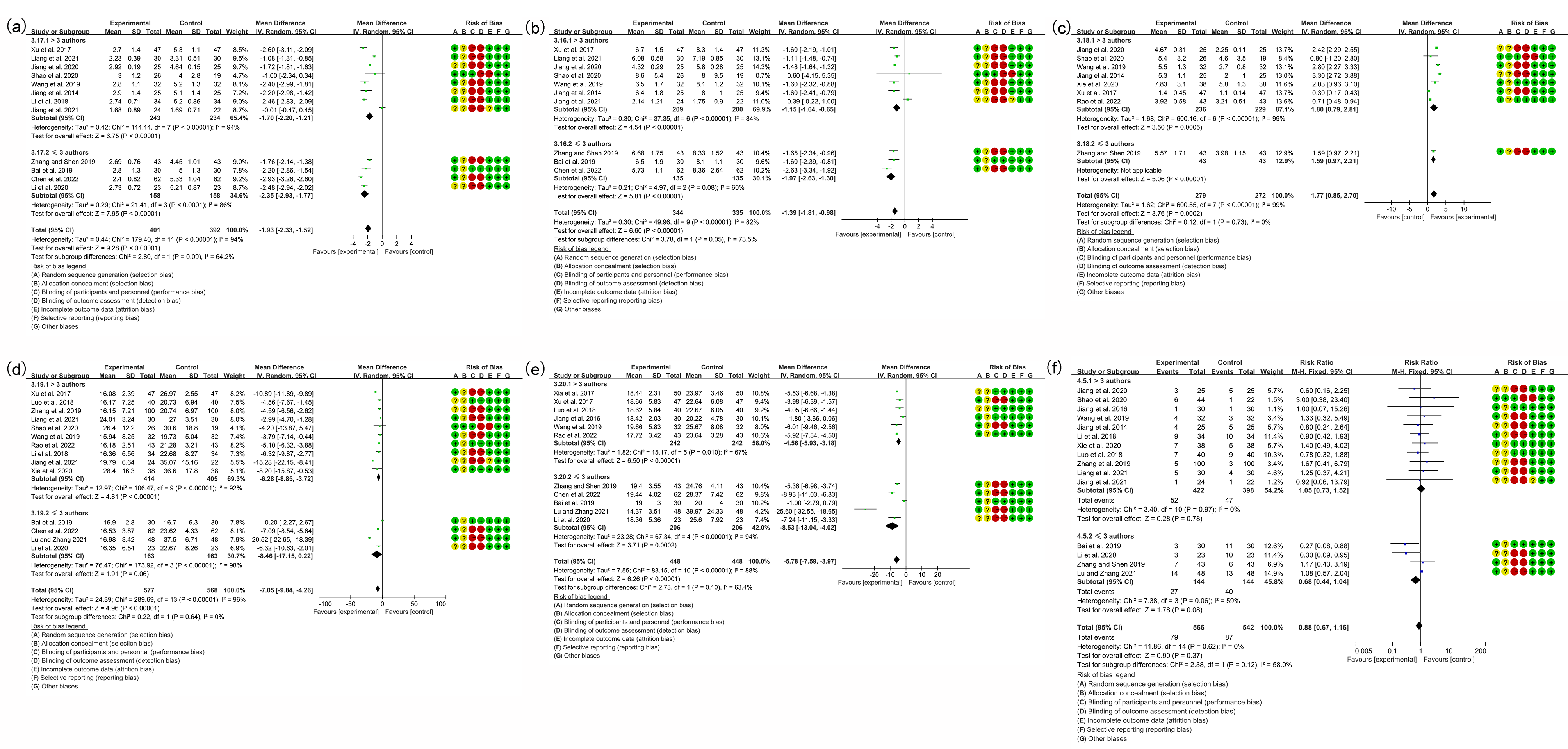

Supplement: Supplementary file 4 [file Image_4.tif]
