# Supplementary material for: A Systematic Review and Meta-Analysis of 19 Randomized Controlled Trials of Iguratimod Combined With Other Therapies for Sjogren’s Syndrome
Source: Front Immunol. 2022 Jul 28;13:924730. doi: 10.3389/fimmu.2022.924730 (PMC9367640; doi:10.3389/fimmu.2022.924730)
Supplement: Supplementary file 5 [file Table_1.docx]

**Table S1.** Search Strategies for Pubmed and Embase

| **PubMed** | (Iguratimod OR Alamode OR T-614 OR C17H14N2O6S OR CAS 123663-49-0 OR IGU OR 3-Formylamino-7-methylsulfonylamino-6-phenoxy-4H-1-benzopyran-4-one)  AND  (Sjogrens Syndrome OR Syndrome, Sjogren's OR Sjogren Syndrome OR Sicca Syndrome OR Syndrome, Sicca OR Primary Sjogren's syndrome)  AND  (random* controlled trial [pt] OR controlled clinical trial* [pt] OR randomized [tiab] OR placebo [tiab] OR drug therapy [sh] OR random* [tiab] OR trial* [tiab] OR group* [tiab])  NOT  (animals [mh] NOT humans [mh]) |
| --- | --- |
| **EMBASE** | 1 'Iguratimod'  2 'Alamode'  3 ('T-614' or 'C17H14N2O6S' or 'IGU').ti,ab.  4 1 or 2 or 3  5 'Sjogren's Syndrome'/exp  6 'Sjogrens Syndrome'  7 'Sjogren Syndrome '  8 'Sicca Syndrome'  9 'Primary Sjogren's syndrome'  10 5 or 6 or 7 or 8 or 9  11 'randomized controlled trial'  12 'single blind procedure' or 'double blind procedure'  13 'crossover procedure'  14 12 or 13 or 14  15 4 and 10  16 15 and 14 |
